# Cholera Morbus

**Published:** 1831

**Authors:** 


### LXXV.

### Cholera Morbus.

57, *Old Steyne, Brighton.*

My dear Sir,

As cholera excites so much attention at present, you may, perhaps, find something in the following notes which may deserve a corner in your useful Journal. On a subject so obscure, one ought to gather *facts* in every direction, and then set to analyzing them. Believe me, my dear Sir,

Yours very truly,

J. T. Todd, M.D.

Remarks on Cholera Morbus. Extracted from a Letter of Mr. R. Herrmann, of Moscow.

1. The fluids voided by stool and vomiting contained, besides water, some acetic acid, a small quantity of osmazome, salivary matter, butyric acid and mucus. They resemble very much gastric juice, but do not contain any free muriatic acid. In the alvine discharges, the quantity of butyric acid is greater than in the fluid voided by vomiting, and they contain, besides some albumen, a fetid, oily matter, and a small admixture of bile.

2. The bile of the cholera patients contains the same ingredients as that of healthy persons; it is, however, more concentrated.

3. The secretion of urine ceases almost entirely during the disease. The urine which first re-appears, when the disease has been overcome, contains less urea, and less of the other solid ingredients, than the urine of healthy persons.

4. The blood undergoes considerable changes during the cholera. According to Mr. Herrmann, the blood of healthy persons contains carbonic and acetic acids, in a free state. The blood of the cholera patients contains much less acetic acid, and the quantity of the crassamentum, relative to the serum, is much greater than in healthy persons; and the increased relative quantity of the crassamentum was found to be in direct proportion to the aggravated nature of the disease.

The blood taken from a patient 2 hours before his death contained 62·5 per cent. crassamentum, and 37·5 per cent. serum of sp. gr. 1·036 re-acting alkaline upon litmus papers. The blood of a healthy person, treated in the same manner, gave 43 per cent. crassamentum, and 57 per cent. serum, of specif. gr. 1·027, reacting acid on test papers.

Mr. H. concludes, from his experiments, that the change of the composition of the blood is effected by a part of its ingredients being abstracted by the discharges by stool and vomiting, and that the blood, by parting with its acetic acid and a part of its watery particles, acquires that greater consistency, and that tendency of separating its fibrine, which is observed during the disease.

Dr. Taenichen, in his numerous dissections, found invariably fibrine separated in the heart, forming polypous masses, partly obstructing the arteries.

5. Mr. H. found the air, immediately surrounding the patient, to contain a substance, which, when deposited upon cooled surfaces, resembled animal mucus. It did not re-act upon test-papers, and was precipitated by sugar of lead and tincture of galls, bearing great analogy to the substance which Moscati separated from infected air.

Mr. H. is of opinion that, at a certain stage of the cholera, a miasm is developed, and that, under a certain predisposition of the constitution, the breathing of air containing the infectious matter communicates the disease. It appears that, in Moscow, three individuals amongst 100 possessed this susceptibility for the disease. The proximate causes of the symptoms appear to consist in too copious secretion of the gastric juice, in a spasmodic obstruction of the absorbents of the digestive canal and the biliary ducts, and in a degeneration of the blood, which, when arrived to a certain height, terminates the life of the patient by impeded circulation. The exciting of copious diaphoresis is the only efficacious remedy against cholera, and no patient recovered in Moscow without this critical secretion.

We are much obliged to Dr. Todd for the foregoing extract; for although we do not attach much importance, in general, to the chemical analysis of fluids, in so acute and rapidly fatal a disease as cholera, yet every positive fact is useful as a datum for future theories. We perceive that the College of Physicians has decided on the *extreme* contagious character of cholera, and has recommended quarantine regulations, as strict as if the PLAGUE were the disease in question. These regulations are on the safe side. They will not invite the disease into this country —nor do we think they will prevent its advent, if we have any clear idea of its nature. TIME will tell. We need scarcely allude to the inane, or rather insane, speculations of Sir Anthony Carlisle. A more direct puff was never sent forth from Warrens manufactory or Ely Place! It is contemptible in the highest degree.

---

